# Supplementary material for: MiR-126 Is an Independent Predictor of Long-Term All-Cause Mortality in Patients with Type 2 Diabetes Mellitus
Source: J Clin Med. 2021 May 28;10(11):2371. doi: 10.3390/jcm10112371 (PMC8198825; doi:10.3390/jcm10112371)
Supplement: Supplementary file 1 [file jcm-10-02371-s001.zip › Table S2.pdf]

Supplemental Table S2. miRNAs expression interaction with antiplatelet therapy.

miRNA	ASA 75 mg	ASA 150 mg	Clopidogrel	p value
**miR-126**	0.53 [0-14972.2]	0.54 [0.011-279.17]	2.54 [0.017-2846.55]	**0.047**
**Let-7e**	0.53 [0.001-4754.24]	0.060 [0.007-119.84]	1.38 [0.005-2069.41]	**0.046**
**miR-223**	7.16 [0.006-106095.21]	9.18 [0.16-1657.74]	23.02 [0.31-1998.91]	0.079
**miR-125a-3p**	0.008 [0.0-24.67]	0.08 [0.0-26.02]	0.023 [0.0-1.206]	0.134

Data are expressed as median and range and compared using Kruskal-Wallis test.
