# Supplementary material for: MiR-126 Is an Independent Predictor of Long-Term All-Cause Mortality in Patients with Type 2 Diabetes Mellitus
Source: J Clin Med. 2021 May 28;10(11):2371. doi: 10.3390/jcm10112371 (PMC8198825; doi:10.3390/jcm10112371)
Supplement: Supplementary file 1 [file jcm-10-02371-s001.zip › Table S3.pdf]

Supplemental Table S3. Multivariate Cox regression model for prediction of long-term all-cause of mortality for miR-126, Let-7e, miR-223 and miR-125a-3p

Variable		HR	95%CI		p-value
			Lower	Upper	
miR-126	**High miR-126**	6.749	2.396	19.011	**0.0003**
	**Age**	1.066	1.016	1.118	**0.009**
	**Gender (male)**	3.290	1.034	10.465	**0.044**
	**history of smoking**	1.858	0.616	5.607	0.271
	**prior ischemic stroke**	4.031	1.288	12.620	**0.017**
	**GFR<30**	9.135	1.421	58.738	**0.020**
let-7e	**High let-7e**	5.231	1.866	14.659	**0.002**
	**Age**	1.062	1.011	1.115	**0.016**
	**Gender (male)**	3.981	1.258	12.604	**0.019**
	**history of smoking**	1.517	0.548	4.201	0.423
	**prior ischemic stroke**	2.256	0.932	7.009	0.068
	**GFR<30**	8.038	1.431	45.149	**0.018**
miR-223	**High miR-223**	2.850	1.080	7.525	**0.034**
	**Age**	1.063	1.012	1.116	**0.014**
	**Gender (male)**	3.729	1.194	11.646	**0.023**
	**history of smoking**	1.646	0.611	4.430	0.324
	**prior ischemic stroke**	2.345	0.846	6.498	0.101
	**GFR<30**	11.602	2.082	64.656	**0.005**
miR-125a-3p	**High miR-125a-3p**	2.784	1.181	6.560	**0.019**

	**Age**	1.059	1.010	1.111	**0.019**
	**Gender (male)**	3.611	1.149	11.351	**0.028**
	**history of smoking**	1.781	0.666	4.759	0.250
	**prior ischemic stroke**	2.126	0.774	5.841	0.144
	**GFR<30**	8.648	1.518	49.277	**0.015**

HR, hazard ratio; MI, myocardial infarction, 95%CI, 95% confidence interval;
