# Supplementary material for: MiR-126 Is an Independent Predictor of Long-Term All-Cause Mortality in Patients with Type 2 Diabetes Mellitus
Source: J Clin Med. 2021 May 28;10(11):2371. doi: 10.3390/jcm10112371 (PMC8198825; doi:10.3390/jcm10112371)
Supplement: Supplementary file 1 [file jcm-10-02371-s001.zip › Table_S1.pdf]

Supplemental Table S1. Patient's characteristics according to miR-126, Let-7e, miR-223 and miR-125a-3p expression subgroup. Comparison of the study groups.

**Patient demographics**	**Low miR-126 N= 150 (59.5%)**	**High miR-126 N= 102 (40.5%)**	**p-value**	**Low Let-7e N= 132 (52%)**	**High Let-7e N= 120 (48%)**	**p-value**	**Low miR-223 N= 150 (59.5%)**	**High miR-223 N= 102 (40.5%)**	**p-value**	**Low miR-125a-3p N= 132 (52%)**	**High miR-125a-3p N= 120 (48%)**	**p-value**
miRNA	0.403 ± 0.506	321.362 ± 1888.45	**<0.000**	0.187 ± 0.202	91.04 ± 495.97	**<0.000**	2.15 ±1.69	1680.95 ± 11411.81	**<0.000**	0.06 ± 0.29	0.81 ± 3.66	**<0.000**
Age (years) mean ± SD	67.23 ± 8.36	66.81 ± 8.82	0.702	67.18 ±8.78	66.92 ± 8.28	0.811	68.10 ±8.47	66.22 ±8.50	0.082	66.77 ±8.26	67.17 ±8.90	0.651
Sex (female) n (%)	65 (56%)	51 (44%)	0.320	56 (48%)	61 (52%)	0.200	48 (41%)	69 (59%)	0.071	63 (56%)	50 (44%)	0.427
Body mass index (BMI)	32.18 ±15.94	30.41 ± 5.16	0.281	32.19 ± 16.93	30.67 ± 5.13	0.347	32.44 ±17.98	30.64 ±5.11	0.709	32.18 ±16.02	30.55 ±5.48	0.173
Hypertension	135 (60%)	92 (41%)	0.560	118 (52%)	109 (48%)	0.699	104 (46%)	124 (54%)	0.883	133 (59%)	91 (41%)	0.308
Dyslipidaemia	126 (60%)	86 (41%)	0.760	109 (52 %)	103 (49%)	0.767	95 (45%)	118 (55%)	0.648	125 (60%)	85 (41%)	0.448
HF	55 (61%)	35 (39%)	0.657	48 (52%)	43 (47%)	0.861	42 (46%)	49 (54%)	0.853	53 (60%)	36 (40%)	0.852
Current smoking	17 (68%)	8 (32%)	0.393	16 (64%)	9 (36%)	0.215	14 (56%)	11 (44%)	0.305	18 (72%)	7 (28%)	0.196
CAD	79 (59%)	56 (42%)	0.836	71 (53%)	64 (47%)	0.790	61 (45%)	75 (55%)	0.914	75 (56%)	59 (44%)	0.436

Prior myocardial infarction	49 (65%)	26 (35%)	0.208	43 (58%)	31 (42%)	0.196	34 (45%)	41 (55%)	0.978	42 (57%)	32 (43%)	0.710
History of smoking	82 (57%)	61 (43%)	0.528	74 (52%)	68 (48%)	0.912	62 (43%)	81 (57%)	0.498	83 (58%)	61 (42%)	0.734
ACS	5 (83%)	1 (17%)	0.268	3 (50%)	3 (50%)	0.755	2 (33%)	4 (67%)	0.314	4 (67%)	2 (33%)	0.572
Stroke	2 (33%)	4 (66.7%)	0.147	2 (33%)	4 (67%)	0.251	3 (50%)	3 (50%)	0.630	2 (33%)	4 (67%)	0.158

**Laboratory data (mean±SD)**	**Low miR-126 N= 150 (59.5%)**	**High miR-126 N= 102 (40.5%)**	**p-value**	**Low Let-7e N= 132 (52%)**	**High Let-7e N= 120 (48%)**	**p-value**	**Low miR-223 N= 150 (59.5%)**	**High miR-223 N= 102 (40.5%)**	**p-value**	**Low miR-125a-3p N= 132 (52%)**	**High miR-125a-3p N= 120 (48%)**	**p-value**
White blood cell count (x109/L)	6.94 ± 1.84	6.87 ± 1.97	0.786	6.90 ± 1.90	6.89 ± 1.93	0.952	6.91 ±1.91	6.88 ±1.90	0.802	6.76 ±1.84	7.09 ±1.97	0.297
Platelets (x109/L)	220.03 ± 62.07	228.13 ± 60.58	0.336	215.69 ± 59.32	230.69 ± 64.4	0.145	214.76 ±59.90	229.40 ±63.05	0.188	218.28 ±63.46	227.73 ±55.98	0.187

Haemoglobin (g/dL)	13.90 ± 1.2	13.48 ± 1.77	0.056	13.90 ± 1.31	13.52 ± 1.62	0.051	13.84 ±1.29	13.63 ±1.60	0.327	13.91 ±1.25	13.52 ±1.77	0.091
High-sensitivity C-reactive Protein (mg/dL)	2.9 ± 2.6	4.19 ± 5	0.080	2.68 ± 2.42	4.18 ± 4.87	**0.039**	2.85 ±2.36	3.82 ±4.60	0.347	2.97 ±3.05	3.97 ±4.59	0.056
Fibrinogen (mg/dL)	139.16 ± 46.97	143.4 ± 44.31	0.499	139.09 ± 46.61	142.32 ± 45.45	0.599	391.49 ±92.90	412.06 ±112.19	0.420	387.87 ±95.59	426.83 ±110.72	**0.017**
Creatinine (mg/dL)	1 ± 0.3	1.02 ± 0.34	0.608	0.98 ± 0.27	1.04 ± 0.36	0.158	1.00 ±0.29	1.01 ±0.34	0.817	0.97 ±0.26	1.07 ±0.38	0.095
HbA1c	6.96 ± 1.26	7.07 ± 1.4	0.535	6.96 ± 1.29	7.06 ± 1.35	0.570	6.88 ±1.31	7.11 ±1.31	0.052	6.88 ±1.24	7.16 ±1.42	0.089
Tch	156.79 ± 36.79	161.90 ± 36.03	0.304	153.9 ± 35.74	163.57 ± 36.80	**0.045**	156.05 ±36.04	160.74 ±36.99	0.186	153.33 ±36.33	166.60 ±36.43	**0.003**

TG	127.30 ± 57.71	141.35 ± 68.97	0.098	122.94 ± 56.46	143 ± 67.63	**0.011**	125.51 ±58.20	138.43 ±65.67	0.091	125.80 ±56.99	144.30 ±69.29	0.055
HDL	47.86 ± 13.16	53.56 ± 45.98	0.167	47.82 ± 13.24	52.64 ± 42.41	0.232	47.85 ±13.66	51.89 ±39.39	0.607	50.31 ±37.88	49.12 ±12.47	0.279
LDL	83.76 ± 31.29	84.27 ± 26.05	0.900	82.12 ± 30.37	85.51 ± 27.92	0.392	83.92 ±30.08	83.76 ±28.79	0.973	81.94 ±29.51	86.99 ±30.05	0.157

Concomitant medications n (%)	Low miR-126 N= 150 (59.5%)	High miR-126 N= 102 (40.5%)	p-value	Low Let-7e N= 132 (52%)	High Let-7e N= 120 (48%)	p-value	Low miR-223 N= 150 (59.5%)	High miR-223 N= 102 (40.5%)	p-value	Low miR-125a-3p N= 132 (52%)	High miR-125a-3p N= 120 (48%)	p-value
ASA 75mg	103 (62%)	63 (38%)	0.175	93 (56%)	73 (44%)	0.090	81 (49%)	86 (52%)	0.168	103 (62%)	63 (38%)	**0.038**
ASA 150mg	21 (66%)	11 (34%)	0.440	18 (56%)	14 (44%)	0.623	14 (44%)	18 (56%)	0.819	20 (63%)	12 (38%)	0.620
Clopidogrel	25 (57%)	28 (53%)	0.580	20 (38%)	33 (62%)	**0.027**	20 (38%)	33 (62%)	0.152	22 (44%)	28 (56%)	**0.030**
ß-blockers	103 (58%)	74 (42%)	0.732	90 (51%)	87 (49%)	0.561	76 (43%)	102 (57%)	0.178	103 (58%)	74 (42%)	0.765
ACE inhibitors	102 (62%)	62 (38%)	0.137	90 (51%)	87 (49%)	0.207	77 (47%)	88 (53%)	0.568	103 (64%)	59 (36%)	**0.033**
Statins	102 (57%)	76 (43%)	0.424	90 (51%)	88 (49%)	0.465	80 (45%)	99 (55%)	0.727	106 (60%)	72 (40%)	0.688
Oral antidiabetic drugs	125 (59%)	86 (41%)	0.876	112 (53%)	99 (47%)	0.343	97 (46%)	115 (54%)	0.677	127 (61%)	81 (39%)	0.060
Calcium channel-blockers	55 (59%)	39 (42%)	0.928	47 (51%)	46 (50%)	0.718	41 (44%)	53 (56%)	0.663	58 (63%)	34 (37%)	0.348
Proton pump Inhibitors	26 (47%)	23 (53%)	0.153	22 (45%)	27 (55%)	0.066	21 (43%)	28 (57 %)	0.363	25 (50%)	25 (50%)	0.058

Data are reported as mean ± standard deviation (SD). Abbreviations: ACE, angiotensin converting enzyme; ACS, acute coronary syndrome; ASA, acetylsalicylic acid; BMI, body mass index; CAD, coronary artery disease; HDL, high-density lipoprotein; HF, heart failure; LDL, low-density lipoprotein; MI, myocardial infarction; miRNA, microRNA; TG, triglycerides
